# Draft Genome Sequence of *Enterococcus faecium* Strain LCT-EF301, Which Shows Changes in Biochemical Metabolism following Space Flight

**DOI:** 10.1128/genomeA.00103-14

**Published:** 2014-03-06

**Authors:** Zhenhong Chen, Yinhu Li, De Chang, Li An, Yinghua Guo, Junfeng Wang, Tianzhi Li, Yajuan Wang, Xiaojun Zhang, Wenkui Dai, Changting Liu

**Affiliations:** a Nanlou Respiratory Diseases Department, Chinese PLA General Hospital, Beijing, China; b BGI-Shenzhen, Shenzhen, China

## Abstract

An *Enterococcus faecium* strain was sent into space on the Shenzhou-VIII mission. After the space flight, the strain *E. faecium* LCT-EF301 was isolated and sequenced based on the changes to its metabolic properties.

## GENOME ANNOUNCEMENT

The *Enterococcus faecium* strain that was loaded on the Shenzhou-VIII spacecraft was isolated from a clinical environment in 2011 (1). After space flight, we obtained and sequenced a mutant strain of *E. faecium* with phenotypic differences in its metabolic patterns compared to those of the ground control strain *E. faecium* LCT-EF90.

Using the HiSeq 2000 sequencing platform (Illumina, San Diego, CA), the genome of *E. faecium* strain LCT-EF301 was determined at the Beijing Genomics Institute (BGI) (Shenzhen, China). Two randomized libraries with different insert sizes (500 bp and 6,000 bp) were constructed from high-quality genomic DNA samples of strain LCT-EF301. The sequence reads from these two libraries were filtered and assembled using SOAP*denovo* (http://soap.genomics.org.cn/). The libraries provided approximately 223-fold coverage of the genome. In total, 14 scaffolds were obtained based on ligation of 117 contigs, and the scaffold N_90_ was determined to be 2,548,875 bp. The sequences of genes, tRNA, rRNA, and tandem repeats were predicted using methods described previously (1). Gene functions were annotated by BLASTp analysis with the Gene Ontology (GO), KEGG, Swiss-Prot, and Clusters of Orthologous Groups (COG) databases.

The draft genome of *E. faecium* LCT-EF301, excluding the gaps, has a total of 2,696,027 bp and a G+C content of 37.98%. The total length of the 2,642 predicted protein-coding sequences (CDSs) is 2,333,853 bp, which results in a coding intensity of 86.57%. Based on the results of the KEGG annotation, 1,460 genes were assigned to 31 functions in the database, including 426 genes in the membrane transport pathway, which is the primary function of the genome. There were 1,548, 1,233, and 1,615 genes identified in the COG, Swiss-Prot, and GO databases, respectively. From the prediction results of RepeatMasker, RepeatProteinMasker, and Tandem Repeats Finder, 50 tandem repeats and 28 minisatellites were identified. The genome also contains 39 tRNA sequences but no rRNA sequences.

### Nucleotide sequence accession number.

This whole-genome shotgun project of *E. faecium* LCT-EF301 has been deposited at DDBJ/EMBL/GenBank under the accession no. APJR00000000. The version described in this paper is the first version.
